# Grand challenges in stroke genomics

**DOI:** 10.3389/fstro.2022.984176

**Published:** 2022-08-24

**Authors:** Myriam Fornage

**Affiliations:** ^1^Brown Foundation Institute of Molecular Medicine, McGovern Medical School, University of Texas Health Science Center at Houston, Houston, TX, United States; ^2^Human Genetics Center, School of Public Health, University of Texas Health Science Center at Houston, Houston, TX, United States

**Keywords:** genomics, stroke, genome-wide association, genetics, omics

Stroke, a leading cause of death, disability and dementia, is a significant public health burden with enormous financial and human costs that are projected to rise over the next decades due to demographic shifts in populations around the globe (Donkor, 2018). Stroke is a complex and heterogeneous disease encompassing multiple etiological subtypes. Recent genetics and omics studies have yielded some exciting novel insights into stroke pathophysiology and the promise of new advances in stroke prevention, diagnosis, treatment, and outcome. In particular, genome-wide association studies (GWAS) have proven an essential tool to identify common variants associated with stroke, thus unveiling previously unsuspected biological mechanisms underlying stroke and stroke subtypes (Neurology Working Group of the Cohorts for Heart and Aging Research in Genomic Epidemiology (CHARGE) Consortium, the Stroke Genetics Network (SiGN), and the International Stroke Genetics Consortium (ISGC), 2016; NINDS Stroke Genetics Network and International Stroke Genetics Consortium, 2016; Malik et al., 2018; Keene et al., 2020). For example, the discovery of a common variant in the *HDAC9* gene as the strongest genetic risk factor for large vessel stroke identified to date has shed light on a potential mechanism related to atherosclerotic plaque vulnerability (Asare et al., 2020). With a growing number of stroke gene discoveries owing to sustained increases in sample sizes and development of high-throughput omics discovery methods, the opportunities for genomic medicine applications in stroke have never been so great. Yet, several enduring challenges in stroke genomics remain to be addressed.

## Increasing diversity in stroke gene discovery

Most stroke risk loci known to date have been identified in populations of European ancestry. With few exceptions (Keene et al., 2020; Kumar et al., 2021), populations of African, Hispanic, and South-Asian ancestry who bear the largest burden of stroke worldwide are underrepresented in current GWAS. There are both scientific and ethical arguments to support the need for greater diversity in stroke genomic studies. Diversity may be defined in terms of ancestral/genetic background, environmental exposures, and socio-cultural/ socio-demographic factors. Increasing ancestral/genetic diversity is essential for the fine-mapping of functional variants (Asimit et al., 2016; Graham et al., 2021). Moreover, while effect size estimates of identified stroke risk loci have generally shown good correlations across ancestries (Malik et al., 2018), there may exist population-specific variants with allele frequencies differing across ancestry groups. Increasing diversity in environmental influences is particularly relevant for omics approaches that may capture the effect of a variety of non-genetic factors, such as lifestyle factors or the built environment (e.g., air pollution), which play a role in stroke etiology. Increasing socio-cultural diversity is important because social and cultural measures of identity, such as self-reported “race” or “ethnicity,” are intricately tied to historical, political and traditional contexts that often shape institutional and structural inequalities and result in health disparities (Mersha and Abebe, 2015; Williams et al., 2016). Identifying and characterizing the effect of genes on stroke risk in the context of environmental and socio-cultural factors is an essential step toward developing accurate and effective tools for precision medicine, such as polygenic risk scores and pharmacogenetic testing. For these tools to equitably benefit people from all ancestral and socio-cultural backgrounds, there needs to be vastly greater representation of global populations and communities in genetics and omics studies of stroke.

## Looking beyond common genetic variation

As next generation sequencing is steadily gaining in throughput and accuracy while simultaneously decreasing costs, well-powered whole genome sequencing (WGS) studies will provide an opportunity to examine the impact of rare and structural variation on stroke risk, which are not well captured with array-based technologies, and to expand genomic coverage for fine mapping of known stroke loci. The first WGS association analysis of stroke identified 5 novel low-frequency variants in a multi-ancestry sample of 6,833 stroke cases and 27,116 controls (66% European ancestry) from the TOPMed consortium (Hu et al., 2022). No rare variants were identified. Compared to the MEGASTROKE GWAS (Malik et al., 2018), this study afforded a greatly expanded genome coverage (40 million vs. 8 million variants tested) but its sample size remained limited, underscoring the need for larger studies. In parallel, WGS resources generated by the TOPMed program are enabling considerable improvements in genotype imputation of rare variants for GWAS, hence enhancing their power, which should facilitate novel gene discoveries (Taliun et al., 2021).

The continuous advances in high throughput biotechnologies are opening new avenues for generating high-dimensional omics data across multiple biomolecular levels, such as transcriptomics, epigenetics, proteomics, and metabolomics, from bulk tissues or at the single-cell resolution. Analyses of single omics data for biomarker and therapeutic target discovery in stroke are underway but remain plagued by small sample sizes, technical and methodological limitations, including different detection platforms (Montaner et al., 2020). Moreover, single omics analyses cannot capture the complex interactions among the diverse omics layers, which underlie the biological processes influencing health and disease. Integrating these various omics data to extract meaningful knowledge about stroke etiology and identify biomarkers and therapeutic targets remains a daunting task owing to the heterogeneity of data types and data structures. Machine learning and deep learning approaches are likely to play a critical role in meeting the challenges of multi-omics data analysis (Li et al., 2022).

## Moving from loci to genes to mechanisms

Although many stroke risk loci have been robustly identified, our understanding of their underlying molecular mechanisms remains limited. Most GWAS discoveries map within non-coding regions of the genome, such as intronic or promoter regions and intergenic regions, which harbor a variety of regulatory sequences. A long-standing challenge in genetic studies is to characterize the functional effects of non-coding variants and assign causality to variants within a discovered GWAS locus. While prediction of variants effects is steadily improving through the application of machine and deep learning methodologies (Zhou and Troyanskaya, 2015), the complex regulatory code of the human genome, which may be cell type-specific, remains poorly understood. Hence, current functional annotations have limited performance and practical application in post-GWAS analyses.

A more recent approach is to leverage information from common genetic effects on gene regulation that can be inferred from mapping quantitative trait loci (QTL) for various molecular features. The most commonly used are QTLs associated with gene expression levels (eQTLs) because comprehensive catalogs have been made available through large consortia resources such as GTEx and eQTLGen (GTEx Consortium, 2020; Vosa et al., 2021), but QTLs for protein and metabolites levels, DNA methylation, and other molecular features are being generated. These data can prove valuable in pinpointing potential causal genes in individual GWAS loci. For example, on chromosome 1q22, *SLC25A44*, encoding a mitochondrial carrier protein, was prioritized using this strategy (Yang et al., 2019). A current limitation is the inadequate representation of a wide variety of tissue sources derived from diverse populations. The development of induced pluripotent stem cells (iPSC) that can be differentiated into specific cell types or even organoids has the potential to ease this constraint (Song et al., 2021). These model systems can be used for scalable experimental introduction of genetic variants using CRISPR/Cas9 technology (Findlay, 2021). Combined with multi-omics and high throughput imaging or other deep-phenotyping techniques, these cellular models provide important tools for in-depth measures of diverse cellular functions. Although the transformative potential of these multimodal cellular approaches to understand stroke mechanisms is likely to be profound, the biological complexity of the disease may still require studies in model organisms. To date, only two genes identified in stroke GWAS have been functionally studied in animal models (Neurology Working Group of the Cohorts for Heart and Aging Research in Genomic Epidemiology (CHARGE) Consortium, the Stroke Genetics Network (SiGN), and the International Stroke Genetics Consortium (ISGC), 2016; Asare et al., 2020). Development of humanized mouse models such as that developed for Alzheimer's Disease (Espuny-Camacho et al., 2017) through transplantation in the mouse brain of human iPSC-derived cortical neurons may provide key insights for disease mechanism and therapeutic discovery.

## Translating genomic discoveries for population health and precision medicine applications

It is widely anticipated that genomic discoveries will have a transformative impact on personal and population health through implementation of tailored medical treatment and prevention strategies (Guttmacher and Collins, 2005). Although translating stroke genomics discoveries into clinical practice remains challenging, several applications show some promise including polygenic risk scores, diagnostic biomarker discovery, pharmacogenomics and drug development.

Polygenic risk scores (PRSs) are estimates of a person's genetic risk for a trait or disease reflecting the cumulative effect of risk alleles across the genome. Their potential clinical applications include early detection of individuals or populations at risk, who could be targeted for lifestyle or therapeutic interventions; refinement of diagnosis; and guidance on therapeutic modalities and patient management. Recently, a PRS for stroke was developed by combining PRSs for each of 19 stroke and stroke-related vascular risk factors into a single meta-genetic risk score (“metaGRS”) (Abraham et al., 2019). In the UK Biobank, the metaGRS was as predictive as or more predictive than any single traditional risk factor, including blood pressure; and individuals in the 0.25% of the metaGRS distribution had a level of risk of ischemic stroke almost as high as that imparted by monogenic stroke (three-fold). In the Atherosclerosis Risk In Communities (ARIC) study, individuals aged 45 years with a high metaGRS had a remaining lifetime risk of stroke of 23% vs. 10% for those with a low metaGRS, corresponding to ~3 years less lived (Thomas et al., 2022). In a retrospective analysis of 5 cardiovascular clinical trials, a stroke PRS developed from 32 genetic variants identified in the MEGASTROKE GWAS was a significant and independent predictor of ischemic stroke risk but not recurrent stroke (Marston et al., 2021). In addition, among patients with atrial fibrillation, those with a high PRS were at significantly elevated risk of developing of stroke despite having a low CHA_2_DS_2_-VASc clinical score, suggesting that the PRS may help in guiding management of a subgroup patients who may benefit from anticoagulation therapy. While the translational applications of PRSs are promising, there currently remains considerable barriers to their widespread clinical deployment and additional progress must be made to achieve an equitable deployment and use of PRSs so as not to exacerbate existing health disparities (Martin et al., 2019). In particular, improvements in PRS development and evaluation are needed. Indeed, most PRS have a much greater predictive power in populations of European ancestry than others, most notably African ancestry (Mars et al., 2022). For example, in the ARIC study, the predictive power of the stroke metaGRS was ten-fold greater in Whites than in Blacks (Thomas et al., 2022). Beyond ancestry biases, there may be other factors that influence performance of PRSs and that remain addressing, including context-dependent effects (e.g., gene-environment interactions, sex-specific effects), stroke subtype specificity, incorporation of rare variants, as well as ways for selecting the best performing PRS. As methods to develop and evaluate PRSs vary extensively, there is a need for standardization and transparency in PRS curation and reporting. To this end, the Polygenic Score Catalog provides an important open resource (Lambert et al., 2021).

Blood-based biomarkers that aid in the diagnosis of several cardiovascular diseases have been available for many years (e.g., D-dimer for venous thromboembolism; troponin for myocardial infarction). For stroke, no such biomarkers are available. Despite longstanding efforts to identify blood-based biomarkers that can distinguish between ischemic and hemorrhagic stroke subtypes, none has yet achieved sufficient sensitivity or specificity for clinical use (Kamtchum-Tatuene and Jickling, 2019). A biomarker panel including Glial fibrillary acid protein (GFAP), retinol binding protein 4 (RBP-4), N-terminal proB-type natriuretic peptide (NT-proBNP) developed in a small sample of patients was shown to distinguishes IS from ICH with moderate accuracy (Bustamante et al., 2021) but larger multicenter studies are warranted. The advent of large omics studies, most notably proteomics studies is likely to accelerate this quest, especially when combined with powerful Mendelian Randomization (MR) techniques (Davey Smith and Hemani, 2014). A recent systematic MR screen of the circulating proteome identified the scavenger receptor class A5 (SCARA5) as a promising biomarker of cardioembolic stroke. Prediction of side-effect profiles showed that it had no predicted adverse side effects, suggesting that it may be prioritized as a new therapeutic target (Chong et al., 2019). Although MR has known limitations, this and other studies suggest that integrating multi-omics data through MR techniques will facilitate drug discovery through identification of new targets, prediction of drug effects, including adverse effects.

Indeed, drug repurposing provides opportunities for stroke genomics applications, because previously approved drugs can be more readily carried forward to clinical practice. Databases such as the DrugBank (Wishart et al., 2006) or the Therapeutic Targets Database (Wang et al., 2020) hold information on immediate biological targets of drugs that can be leveraged for translation of genetic discoveries. For example, linking findings from the MEGASTROKE GWAS, it was shown that identified stroke risk loci were significantly enriched for genes encoding targets of alteplase, an approved thrombolytic drug, and cilostazol, an antiplatelet agent approved in Asia (Malik et al., 2018). In MR analyses using genetic proxies for antihypertensive drug targets, calcium channel blockers, but not β-blockers, were associated with lower risk of stroke, especially small vessel stroke and white matter hyperintensities on neuroimaging. Hence, calcium channel blockade may represent a promising strategy for preventing cerebral small vessel disease (Georgakis et al., 2020).

Pharmacogenomics, the study of the contribution of genomic variation on individual responses to drug treatment, is an active area of research in stroke. A prime example is that of clopidogrel, a commonly prescribed antiplatelet drug, which exhibits a wide variation in patient responsiveness (Angiolillo et al., 2007). Carriers of specific loss-of-function alleles in *CYP2C19* have a significantly reduced metabolism of clopidogrel into its active form and, when treated with clopidogrel, have an increased risk of major adverse cardiovascular events, especially after percutaneous coronary intervention (PCI) (Mega et al., 2010). Clinical trials conducted to date show some promises of a genotype-informed antiplatelet selection after PCI compared with a conventional clopidogrel therapy (Pereira et al., 2020; Wang et al., 2021). These include an improved efficacy (reduction in ischemic events) and reduced toxicity (reduction in bleeding complications), and a greater cost-effectiveness (Liu et al., 2020; Narasimhalu et al., 2020).

## Understanding post-stroke outcomes and VCID

While tremendous progress has been made in understanding stroke risk, the functional and cognitive outcomes after stroke are only now being tackled (Lee et al., 2021). Critical to meeting this challenge is the development of informative phenotypes and endophenotypes from the acute, subacute and chronic phases. These phenotypes must capture the dynamic processes that underlie the spectrum of injury and recovery after stroke. The change in National Institutes of Health Stroke Scale (NIHSS) during the first 24 h post stroke (ΔNIHSS_24h_) has been used as a measure of evolution of neurological deficits in the acute phase. A GWAS of ΔNIHSS_24h_ has identified 7 genetic loci that harbor genes influencing excitotoxicity (Ibanez et al., 2022). Neuroimaging endophenotypes that capture hemorrhagic transformation or edema formation are also of great interest. Longer term outcome using the modified Rankin Scale (mRS) have also been used in GWAS studies (Mola-Caminal et al., 2019; Soderholm et al., 2019). Future progress will require large standardized and harmonized datasets. The Global Alliance of Acute and Long-Term Outcome is an initiative of the International Stroke Genetics Consortium (ISGC) that provides recommendations and leadership in this area. Post-stroke cognitive impairment and dementia (PSCID) is an important area of opportunities for genomic studies. The DISCOVERY study (Determinants of Incident Stroke Cognitive Outcomes and vascular Effects on Recovery) a large collaborative network among 30 sites will address the critical need for understanding the factors that influence PSCID risk (Rost et al., 2021). Because injury and recovery after stroke are influenced by dynamic processes controlled by changes in gene expression, analyses of omics data that are plastic and sensitive to interventions and changes overtime are particularly well suited to identifying key genes and biological pathways for resilience and recovery after stroke.

## Conclusion

In conclusion, genomics has the potential to revolutionize stroke diagnosis, prevention, treatment, and outcome. Rapid advances in stroke genomics are fueled by investments in technologies and population resources that are brought together in large national and international programs and collaborations, such as NeuroCHARGE and the ISGC, and facilitated by the wide sharing of data through open portals, including the Cerebrovascular Disease Knowledge Portal (Crawford et al., 2018). The contribution of biobanks, including the UK Biobank is likely to accelerate progress. Novel methods that exploit multi-omics and machine learning hold great promise to discovering novel pathways for stroke risk and outcome.

## Author contributions

The author confirms being the sole contributor of this work and has approved it for publication.

## Conflict of interest

The author declares that the research was conducted in the absence of any commercial or financial relationships that could be construed as a potential conflict of interest.

## Publisher's note

All claims expressed in this article are solely those of the authors and do not necessarily represent those of their affiliated organizations, or those of the publisher, the editors and the reviewers. Any product that may be evaluated in this article, or claim that may be made by its manufacturer, is not guaranteed or endorsed by the publisher.
